# The Role of Mitophagy in Ischemic Stroke

**DOI:** 10.3389/fneur.2020.608610

**Published:** 2020-12-23

**Authors:** Ziqi Shao, Shanshan Dou, Junge Zhu, Huiqing Wang, Dandan Xu, Chunmei Wang, Baohua Cheng, Bo Bai

**Affiliations:** ^1^Cheeloo College of Medicine, Shandong University, Jinan, China; ^2^Neurobiology Institute, Jining Medical University, Jining, China

**Keywords:** molecular mechanisms, signaling pathway, ischemia-reperfusion injury, ischemic stroke, mitophagy

## Abstract

Mitochondria are important places for eukaryotes to carry out energy metabolism and participate in the processes of cell differentiation, cell information transmission, and cell apoptosis. Autophagy is a programmed intracellular degradation process. Mitophagy, as a selective autophagy, is an evolutionarily conserved cellular process to eliminate dysfunctional or redundant mitochondria, thereby fine-tuning the number of mitochondria and maintaining energy metabolism. Many stimuli could activate mitophagy to regulate related physiological processes, which could ultimately reduce or aggravate the damage caused by stimulation. Stroke is a common disease that seriously affects the health and lives of people around the world, and ischemic stroke, which is caused by cerebral vascular stenosis or obstruction, accounts for the vast majority of stroke. Abnormal mitophagy is closely related to the occurrence, development and pathological mechanism of ischemic stroke. However, the exact mechanism of mitophagy involved in ischemic stroke has not been fully elucidated. In this review, we discuss the process and signal pathways of mitophagy, the potential role of mitophagy in ischemic stroke and the possible signal transduction pathways. It will help deepen the understanding of mitophagy and provide new ideas for the treatment of ischemic stroke.

## Introduction

Stroke refers to a group of diseases that cause brain tissue damage due to the sudden rupture of blood vessels or the blockage of blood flow into the brain, including hemorrhagic and ischemic stroke. It has the characteristics of high morbidity and high mortality. Ischemic stroke accounts for ~87% of the total number of stroke patients (1). Ischemic stroke refers to a type of disease in which brain tissue necrosis is caused by a narrowing or occlusion of the blood supply arteries (carotid and vertebral arteries) of the brain and insufficient blood supply to the brain. At present, thrombolysis is considered to be the most important method for the treatment of ischemic stroke (2). However, due to the limitations of current thrombolytic therapy such as an optimal treatment time window of only 4.5 h (3), enhancing the self-resistance and protection of neurons has become the focus which has attracted more researchers (4).

Mitochondria are where the oxidative metabolism of eukaryotes takes place, and the major producers of intracellular reactive oxygen species (ROS). They can also regulate membrane potential and control programmed cell death (5). Based on the complex structure and important functions of mitochondria, they are closely related to many diseases including ischemic stroke. When ischemic stroke occurs, the dynamic balance maintained by mitochondria is broken, and related signaling pathways are activated, which lead to cascade damage to nerve cells (6). In the ischemic period of ischemic stroke, mitochondria cannot synthesize enough ATP or cause energy disorders due to lack of oxygen and energy substances. During the reperfusion period, the increase of ROS and mitochondrial membrane lipid peroxidation lead to oxidative stress damage. Increased ROS also disrupts the calcium pump on the mitochondrial membrane, which induces calcium overload and inflammatory response. In addition to these pathological changes, cell death in ischemic stroke including apoptosis and autophagy are all related to the loss of mitochondrial function (7). Therefore, the research on the correlation between mitochondria and ischemic stroke can not only fully explain the mechanism of the occurrence and development of ischemic stroke, but also provide potential guidance and help for the innovative treatment of ischemic stroke.

Autophagy is a biological process in which organelles and proteins are degraded by lysosomes in eukaryotic cells (8). One of the main functions of autophagy is to keep cells alive when they are threatened by stressful death (9). This is an important evolutionary conservation mechanism for eukaryotic cells to maintain homeostasis and achieve renewal (10). Although autophagy in a broad sense includes macroautophagy, microautophagy and chapeon-mediated autophagy (11), it is commonly referred to as macroautophagy. Mitophagy is a type of macroautophagy by which cells selectively clear impaired or dysfunctional mitochondria through the mechanism of autophagy (12). It plays an important role in mitochondrial quality control and cell survival (13). More and more studies have shown that mitophagy is associated with neurodegenerative diseases such as Parkinson's disease (PD), Alzheimer disease (AD) and Huntington's (HD) and brain injury (14). Although there have been studies showing that mitophagy is closely related to ischemic stroke (4, 15), the exact roles of mitophagy still need to be studied further. In this review, we focus on the research progress in the occurrence and regulation of mitophagy in ischemic stroke.

## Mitochondrial Dynamics and Mitophagy

### The Molecular Mechanism of Mitochondrial Dynamics

**Figure 1:**
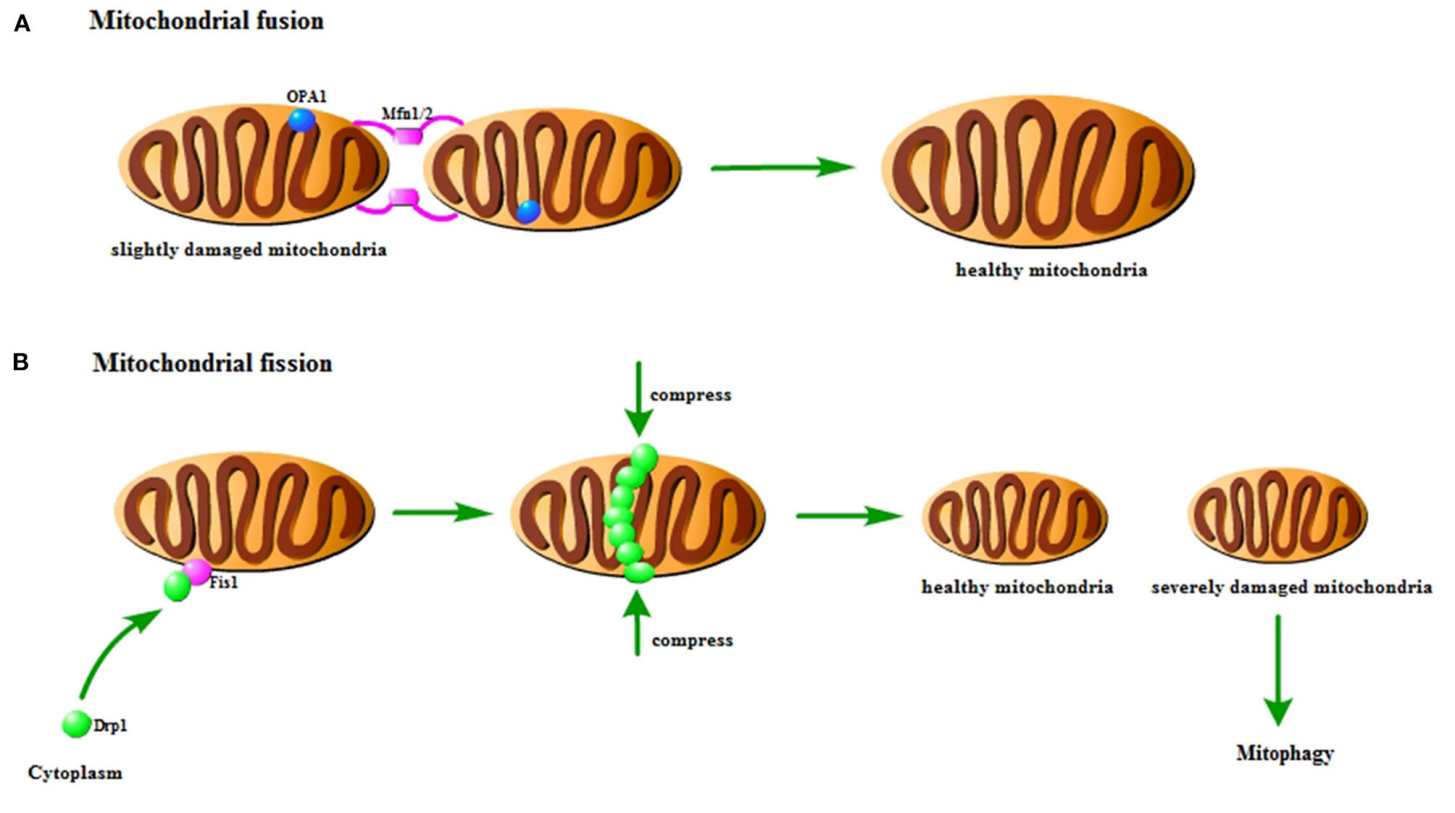
Pattern diagram of mitochondrial fusion and fission. **(A)** Mitochondrial fusion. The Mfn1/2 interaction causes the outer membrane to fuse, and then the Opa1 interaction causes the inner membrane to fuse. It could repair slightly damaged mitochondria. **(B)** Mitochondrial fission. Fis1 on the mitochondria recruits Drp1 to the mitochondria to form a finger ring structure, which squeezes and ruptures the mitochondria. It could distribute the damaged components of mitochondria to the offspring. The severely damaged mitochondria will be cleared by mitophagy.

Mitochondria are highly dynamic organelles that adapt to various stress conditions to meet the energy metabolism and other biological needs through continuous fusion and fission to change their shape (16). This biological process is called mitochondrial dynamics, which is an important basis for maintaining cell homeostasis (17). Mitochondrial fusion is a multi-step process in a certain order: (1) mitochondrial trans-tethering; (2) mitochondrial outer membrane fusion; (3) mitochondrial inner membrane fusion (16). The fusion process of mitochondria is mainly completed by activating three GTPases: mitofusins 1(Mfn1), mitofusins 2 (Mfn2) and optic atrophy 1 (OPA1) (18). Among them, Mfn1 and Mfn2 mainly mediate mitochondrial outer membrane fusion, and OPA1 mainly participates in the mitochondrial inner membrane fusion process (19). The fission process is mainly mediated by dynamin-related protein 1 (Drp1) and fission protein 1 (Fis1) (18). Under the stimulus, Fis1 mediates the translocation of Drp1 in the cytoplasm to the outer mitochondrial membrane. Drp1 accumulates at the mitochondrial fission site to form a “ring” structure and then combines with Fis1 to form a complex, which is gradually compressed until the mitochondria ruptures. Finally, two independent mitochondria are produced (20) (Figure 1).

Disturbance of mitochondrial dynamics is an important phenomenon in cerebral ischemia/reperfusion injury. Studies have found that there is a loss of OPA1 complex during reperfusion (21). In the rat model of cerebral ischemia/reperfusion injury, the expression of Mfn2 in the cerebral cortex is significantly reduced (22). Research has indicated that activating Mfn1 could reduce cerebral ischemia/reperfusion injury (23). In addition, the fission of mitochondria in the hippocampus is found to be activated, and the mitochondria become more and more fragmented with time (24). Previous studies have shown that inhibiting Drp1-dependent mitochondrial fission could protect against cerebral ischemia/reperfusion injury (25). Similarly, the inhibition of Fis1 could also achieve the protective effect (26).

### Interplay Between Mitochondrial Dynamics and Mitophagy

Mitochondrial fusion could repair slightly damaged mitochondria. And mitochondrial fission could not only achieve normal number of proliferation, but also selectively distribute the damaged components of mitochondria to the offspring, which result in healthy mitochondria and severely damaged mitochondria (27). The membrane potential of severely damaged mitochondria cannot be restored. Therefore, the severely damaged mitochondria are unable to participate in fusion, which will be cleared through mitophagy (28). The interaction and mutual regulation between mitochondrial dynamics and mitophagy are important mechanisms for maintaining mitochondrial homeostasis and ensuring mitochondrial quality. In the early stage of ischemia, Drp1-dependent mitophagy is found to contribute to the clearance of damaged mitochondria (29). Studies have shown that mitochondrial fragmentation could regulate mitophagy and apoptosis in cerebral ischemia/reperfusion injury (30). It is also found that OPA1 and Mfn2 are reduced in cerebral ischemia, thereby inducing mitophagy (31). These findings indicate that mitochondrial dynamics is closely related to mitophagy in cerebral ischemia/reperfusion injury.

### Mechanisms and Regulatory Pathways of Mitophagy

#### The Process of Mitophagy

The process of mitophagy is similar to ordinary autophagy. First, permeability changes occur after mitochondria are damaged, which leads to mitochondrial depolarization, and induces the activation of mitophagy-related proteins. Then, the isolation membrane wraps the damaged mitochondria and forms mitophagosomes. After the formation of mitophagosomes, fusion with lysosomes to form mitolysosomes results in the degradation of damaged mitochondria. This process requires the participation of microtubule-associated protein light chain 3 (LC3) and the linker proteins p62, NBR1 and optineurin connecting mitochondria and LC3. In addition, Nix/BNIP3, FUNDC1 also play an important role in this process (32).

#### Mitophagy Signaling Pathway

**Figure 2:**
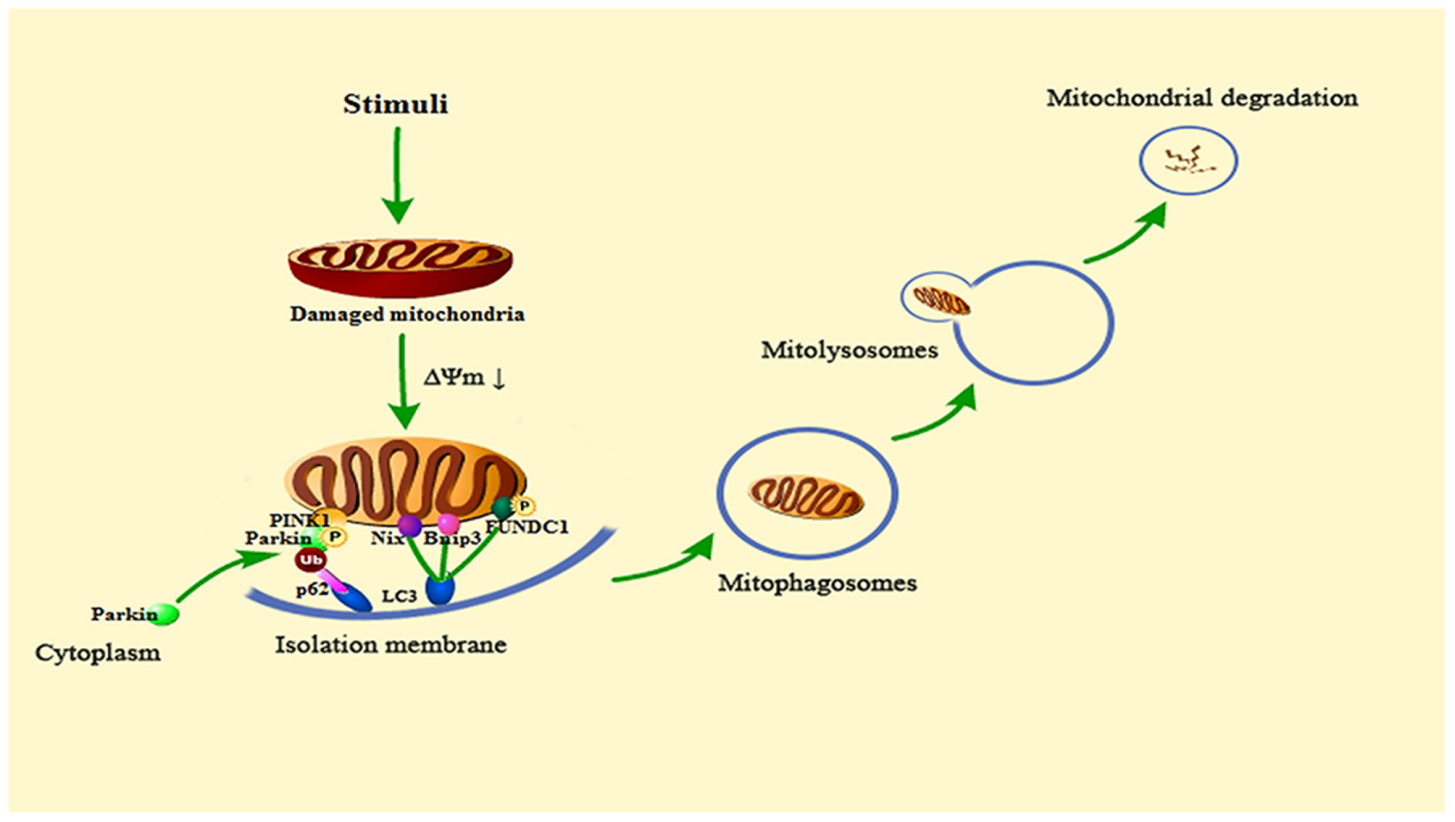
Summary of the mitophagy signaling pathway. When the cell is stimulated, the mitochondria will be damaged. Then the mitochondrial transmembrane potential (ΔΨm) drops, and mitophagy will be activated. PINK1 accumulates on the outer mitochondrial membrane, phosphorylates Parkin and recruits Parkin from the cytoplasm to the mitochondria. Activated Parkin can ubiquitinate the voltage-dependent anion channels 1 (VDAC1) of damaged mitochondria, Then, Parkin is recognized and bound by the signal adaptor protein p62/SQSTM1. P62 could recruit ubiquitinated substances into autophagosomes by binding to microtubule-associated protein light chain 3 (LC3). Bnip3, Nix and phosphorylated FUNDC1 can also connect to LC3 to promote mitophagy. Subsequent mitophagosomes are formed, which bind to lysosomes, and finally lyse damaged mitochondria.

The mechanisms of mitophagy in cells mainly include Parkin-dependent pathways and Parkin-independent pathways. There are multiple signals involved in the regulation of mitophagy, as showed in Figure 2. The current review focuses on PINK1/Parkin, BNIP3/NIX, and FUNDC1 pathway.

#### Parkin-Dependent Mitophagy Pathway

At the beginning of the 21st century, the laboratory of Richard Youle found that Parkin, an E3 ubiquitin ligase, could mediate mitochondria to be wrapped by autophagosomes, which creates a new breakthrough for the study of mitophagy (33). Then, subsequent research found that PINK1 (phosphatase and tensin homolog (PTEN)-induced putative protein kinase (1), a serine/threonine kinase, is located upstream of Parkin (34–36). PINK1 can phosphorylate Parkin and promote the translocation of Parkin from cytoplasm to mitochondria (37). PINK1/Parkin is the clearest pathway for the research of mitophagy.

In healthy mitochondria, the PINK1 protein exists on the outer mitochondrial membrane. It can be introduced into the mitochondrial membrane space and degraded by proteases on the inner mitochondrial membrane to maintain the basic level (38). However, when mitochondria are damaged and depolarized, their ability to degrade PINK1 is weakened, and PINK1 can stably exist on the outer mitochondrial membrane (39). Then, it can phosphorylate both ubiquitin and Parkin to recruit Parkin from the cytoplasm to the outer mitochondrial membrane (40). The stability of PINK1 on the outer mitochondrial membrane is necessary for Parkin to be recruited to damaged mitochondria and to stimulate mitophagy. Activated Parkin can ubiquitinate the voltage-dependent anion channels 1 (VDAC1) of damaged mitochondria. Then, Parkin is recognized and bound by the signal adaptor protein p62/SQSTM1. P62 could recruit ubiquitinated substances into autophagosomes by binding to LC3, which ultimately leads to mitochondria degraded by lysosome (41–43). In the penumbra of rat cortex, fluorescence results show that PINK1 accumulates on the outer mitochondrial membrane and Parkin mitochondrial translocation occurs following ischemia and reperfusion, and the levels of other related autophagy proteins such as LC3 and Beclin1 are elevated. These results may demonstrate that mitophagy is activated in ischemic stroke (44).

#### Parkin-Independent Mitophagy Pathway

Different from PINK1/Parkin-mediated mitophagy, some proteins on the outer mitochondrial membrane can directly recognize and bind LC3. Then, targeted mitochondria are connected with autophagic vesicles, which directly induces mitophagy (45). In this review, we focus on the Nix/Bnip3 and FUNDC1 signaling pathways. They are the most important pathways in Parkin-independent mitophagy.

#### Nix/Bnip3-Mediated Mitophagy Pathway

Bnip3 and Nix (BNIP3L) have about 56% amino acid sequence identity, and both are located in mitochondria (46). Bnip3 is a pro-apoptotic mitochondrial protein. And it is also an important participant in the process of autophagy and even mitophagy (47, 48). Bnip3 is the target gene of HIF1α (hypoxia inducible factor 1α). Under hypoxic conditions, Bnip3 could activate autophagy (49). Similarly, in ischemia and reperfusion, Bnip3 could clear damaged mitochondria by activating mitophagy (49, 50). In the process of red blood cell maturation and development, Nix is essential for the removal of mitochondria. Mitochondrial depolarization, increased production of ROS and hypoxia can induce Nix to regulate mitophagy (51, 52). Bnip3 and Nix could competitively bind to the anti-apoptotic Bc1-2, dissociate the Bc1-Beclin1 complex and release Beclin1, and then activate autophagy and mitophagy (53). In cerebral ischemia/reperfusion injury, Bnip3 and Nix could participate in the induction of mitophagy (54). However, Studies have found that the up-regulation of Nix cannot restore the mitophagy defect caused by Bnip3 deletion in stroke (55). This may indicate that Bnip3 could activate excessive mitophagy leading to cell death, whereas Nix may only regulate basal levels of mitophagy in physiological conditions. Therefore, Bnip3 may be a potential target for the treatment of ischemic stroke in the future.

#### FUNDC1-Mediated Mitophagy Pathway

FUNDC1 is a tertiary transmembrane protein on the outer mitochondrial membrane. The FUNDC1 protein contains a N-terminal LC3 interaction region motif, which plays an important role in mitophagy (56). Under normal conditions, FUNDC1 can stably exist on the outer mitochondrial membrane without mediating mitophagy. When mitochondria are damaged or dysfunctional, the affinity of FUNDC1 and LC3 will increase. Then FUNDC1 can be dephosphorylated to activate, which will induce mitophagy (57). In myocardial ischemia/reperfusion, studies have found that hypoxic preconditioning could induce FUNDC1-dependent mitophagy to resist ischemia/reperfusion injury (58). However, some studies have shown that inhibition of mitophagy mediated by the mTORC1-ULK1-FUNDC1 pathway can protect myocardium from ischemia/reperfusion injury (59). This indicates that similar mechanisms may exist in cerebral ischemia/reperfusion.

### Roles of Mitophagy in Ischemic Stroke

The brain is the main organ of energy metabolism, and the content of mitochondria in the brain is much higher than other tissues (60). Even if short-term ischemia and hypoxia may cause serious injury to the brain, overproduction of free radicals and calcium overload after reperfusion also cause more extensive damage to cells and tissues of the brain. Current research suggests that cerebral ischemia/reperfusion injury is related to the production of free radicals, excitatory amino acid toxicity, mitochondrial dysfunction, and activation of apoptosis-related genes (7, 61–63). Mitochondrial dysfunction is an important part of cerebral ischemia/reperfusion injury, and mitophagy plays a significant role in it. Elimination of abnormal mitochondria through mitophagy is essential for maintaining normal cell function in ischemic stroke.

More than 50 years ago, transmission electron microscopy first discovered the existence of autophagosomes (64–66). As the research on autophagy gets deeper, research methods on autophagy are becoming more and more abundant. Accumulated evidence indicated that autophagy is activated in brain tissue in many nervous system diseases including ischemic stroke (67–70). And mitophagy, as a special type of autophagy, is also found to be activated in ischemic stroke (44). In cerebral ischemia/reperfusion injury, early ischemia and hypoxia damage the structure and function of mitochondria in brain cells. After the oxygen supply and energy supply are restored, the mitochondrial permeability transition pore opens (mPTP) and the mitochondrial membrane potential (MMP) decreases, and then mitochondrial damage is followed by activation of mitochondrial autophagy (71).

Like macroautophagy, we are not sure whether mitophagy is beneficial or harmful in ischemic stroke. The degree of mitochondrial permeability transition (MPT) may play an important role in it (72). Under mild starvation or hypoxia, limited MPT can only damage a small part of mitochondria and then activate mitophagy. At this time, mitophagy not only provides energy by degrading proteins, but also removes damaged mitochondria to protect the cells. In the case of severe starvation or hypoxia, mitophagy is insufficient to clear the damaged mitochondria, and then the autophagy system will be overloaded, which will activate apoptosis-related regulatory proteins and promote the occurrence of apoptosis. When excessive stress causes drastic changes in the MPT of all mitochondria in the cell, cell necrosis will occur.

### Neuronal Mitophagy in Ischemic Stroke

#### Enhancing Mitophagy Reduced Cerebral Ischemic-Reperfusion Injury

Studies have shown that rapamycin could protect against cerebral ischemia/reperfusion injury by activating mitophagy and reducing mitochondrial dysfunction in transient middle cerebral artery occlusion (tMCAO) model. And these protective effects can be reversed by 3-methyladenine, an autophagy inhibitor (73). There are similar findings in the oxygen-glucose deprivation model of hippocampal neurons (74). And activating mitophagy to clear excessively aggregated and damaged mitochondria can reduce neuronal damage caused by cerebral ischemia/reperfusion injury (75–79). Knockout of the mitophagy-related gene Bnip3L could aggravate cerebral ischemia/reperfusion injury, and overexpression of this gene could rescue (54). Studies have also found that activating Parkin-dependent mitophagy could inhibit the activation of NLRP3 inflammasome to reduce cerebral ischemia/reperfusion injury (80). Activation of PARK2-mediated mitophagy may be the basis for protecting endoplasmic reticulum stress in cerebral ischemia/reperfusion injury (81) and extending the limited reperfusion window (82).

#### Inhibiting Mitophagy Reduced Cerebral Ischemic-Reperfusion Injury

However, there are still some studies showing that inhibiting excessive mitophagy can play a protective role in cerebral ischemia/reperfusion injury. In the middle cerebral artery occlusion model (MCAO) of ischemic stroke, studies have found that inhibiting mitophagy can protect against cerebral ischemia/reperfusion injury (83). In the oxygen glucose deprivation model of SH-SY5Y cells, inhibition of mitochondrial calcium uniporter and the influx of Ca^2+^ into mitochondria could inhibit excessive mitophagy and reduce neuronal damage (84). And inhibiting Peroxynitrite-mediated mitochondrial activation could reduce neuronal damage in ischemic stroke (85, 86). In neuronal death caused by chronic cerebral hypoperfusion, it is also found that inhibiting excessive mitophagy could exert neuroprotective effects (87). Similarly, inhibition of AMPK-mediated mitophagy could reduce the ischemic and hypoxic damage of neurons in ischemic hypoxic encephalopathy (88).

Although the differences in the above results may be caused by different ischemia or reperfusion time, different cell types, or even different experimental environments, it is undeniable that mitophagy plays an important role in the pathological mechanism of cerebral ischemia/reperfusion injury. When it is at the basic level, it may be beneficial to cell homeostasis and neuron survival. But it can be harmful when it reaches excess or deficiency. Therefore, the role of mitophagy in ischemic stroke should be studied more deeply to provide novel ideas and targets for clinical treatment.

### Glial Mitophagy in Ischemic Stroke

Glial cells can not only support, nourish, and protect neurons, but also receive signals from neurons. Through their own function, metabolism and morphological changes, glial cells could affect the function and activity of neurons (89). After cerebral ischemic injury, glial cells are activated. In the early stage of cerebral ischemic injury, the activation of glial cells can play a certain neuroprotective effect, but the excessive activation of glial cells can produce a series of inflammatory factors or mediators to mediate neuronal degeneration (90). Previous study has found that hypoxia and reoxygenation of astrocytes caused increased mitochondrial fission and mitophagy (91). In the rat cortex after cerebral ischemia and reperfusion, the activation of mitophagy in astrocytes is also found (83). There are many studies on glial cells autophagy in ischemic stroke while mitophagy-related reports are few. More investigations on astrocytes or microglial mitophagy are needed.

### Interplay Between Mitophagy and Other Cellular Processes in Ischemic Stroke

**Figure 3:**
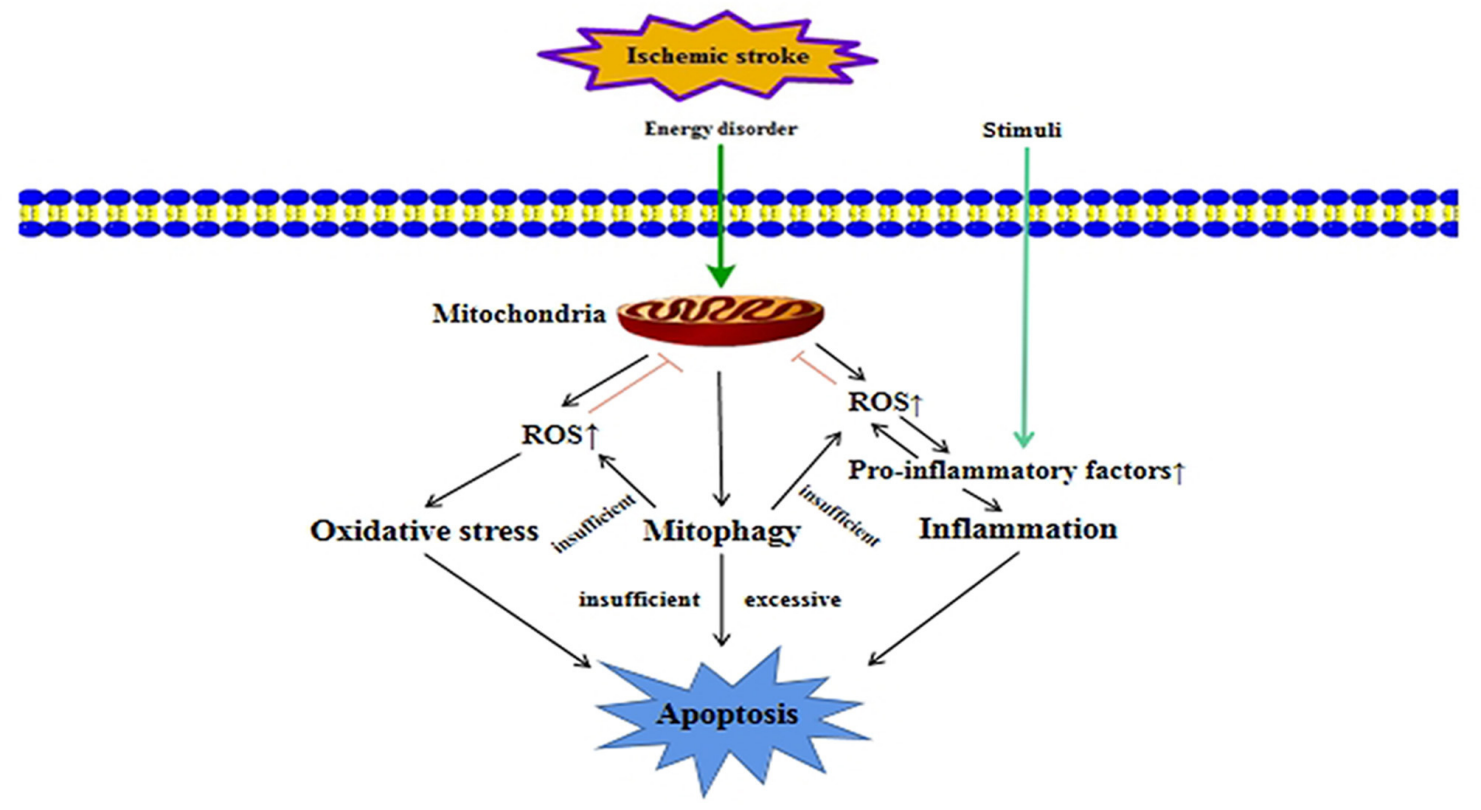
Crosstalk between mitophagy and other cellular processes in ischemic stroke. In ischemic stroke, ischemia and hypoxia can cause energy disorders, which will damage mitochondria. Mitophagy is activated when mitochondria are damaged. When mitophagy is insufficient or excessive, it can lead to apoptosis. Mitochondrial damage will also release a large amount of ROS, leading to oxidative stress damage. At the same time, excessive ROS will promote the release of pro-inflammatory factors, which lead to inflammation. When mitophagy is not enough to clear over-produced ROS, it will aggravate oxidative stress damage and inflammation, which could promote apoptosis.

Mitochondria are the most important organelles involved in energy metabolism in cells. They play a key role in cell signal transduction, free radical generation and apoptosis induction, and determine the survival and death of cells. In the occurrence and development of ischemic stroke, mitophagy is closely related to many biological processes in the cell, such as apoptosis, oxidative stress, and inflammation (Figure 3). These biological processes interact with mitophagy to regulate mitochondrial quality, which could affect the survival and death of nerve cells.

#### Mitophagy and Apoptosis

Unlike mitophagy, apoptosis only has a one-way effect on cell fate. It removes aging and severely damaged cells through a programmed death regulation mechanism. Mitophagy and apoptosis have obvious differences in biochemical metabolic pathways and morphology, but they are functionally antagonistic, coordinated, and promote each other, and participate in the regulation of mitochondrial quality (92–95).

Mitophagy and apoptosis are mostly mutually antagonistic to achieve mutual regulation. Under stress conditions such as ischemia and hypoxia, the phosphorylation of the anti-apoptotic protein Bcl-2 could destroy its binding to autophagy-related protein Beclin1 and activate mitophagy. At the same time, Bcl-2 could prevent the release of pro-apoptotic proteins by maintaining the integrity of the mitochondrial membrane, which finally inhibits the occurrence of cell apoptosis (96). When the cell is under continuous and severe stress, apoptosis can inhibit the occurrence of mitophagy by cleaving the key autophagy protein Beclin1 by activated Caspase and avoid the mitochondrial dysfunction caused by excessive mitophagy (97, 98). However, other studies have shown that mitophagy and apoptosis are functionally coordinated and mutually promoted. Excessive induction of mitophagy can cause the leakage of cathepsin and other hydrolytic enzymes in lysosomes or autophagic lysosomes, and promote the occurrence of apoptosis (99).

Studies have reported that activating mitophagy could inhibit cell apoptosis in ischemic stroke. During the reperfusion phase, mitophagy could inhibit neuronal apoptosis by removing damaged mitochondria (100), and remote ischemic post conditioning could promote Parkin/DJ-1-mediated mitophagy to attenuate apoptosis in MCAO rats (101). Similarly, enhancing Parkin /PINK1-mediated mitophagy could inhibit apoptosis caused by cerebral ischemia/reperfusion injury in hippocampal neurons (74). However, there are still some studies indicating that inhibiting excessive mitophagy can reduce apoptosis. Mitophagy-related protein Bnip3 and Nix could induce excessive mitophagy to promote cell apoptosis in ischemic stroke (55). And inhibition of PINK1/Parkin-mediated mitophagy could reduce the number of apoptotic cells in the cortex of the model group in cerebral ischemia/reperfusion injury (86).

#### Mitophagy and Oxidative Stress

Oxidative stress is caused by free radicals to produce oxidative damage to deoxyribonucleic acid (DNA), lipids and proteins, which leads to aging and neurodegenerative diseases. The oxidation of proteins, lipids and DNA are all related to ROS (102). ROS can lead to mitochondrial lipid peroxidation, membrane potential collapse and ATP synthesis disorder. Mitochondria are the main source of ROS production (103). Mitophagy is closely related to oxidative stress. And it also plays a dual role in oxidative stress.

Under physiological conditions, low levels of ROS can usually be degraded by some antioxidant enzymes or substances. Maintaining the balance between ROS production and degradation is very important for the normal physiological functions. If the anti-oxidant substances in the cell cannot effectively degrade ROS, it will make ROS accumulate in the cell and then cause oxidative stress (104). Excessive ROS and other substances can preferentially activate mitophagy, allowing it to selectively degrade damaged mitochondria, which could reduce damage to cells (105). There are studies showing that activating mitophagy could reduce oxidative stress damage in ischemia/reperfusion injury. In renal ischemia/reperfusion injury, ischemic preconditioning can protect mitochondrial function by activating mitophagy and inhibit the production of ROS (106), and activation of ROS-dependent autophagy promotes the survival of liver endothelial cells in liver ischemia/reperfusion injury (107). Also, in cerebral ischemia/reperfusion injury, enhancing mitophagy could reduce excessive accumulation of ROS (74). However, mitophagy also can directly induce the death of oxidized cells. In myocardial ischemia/reperfusion injury, inhibiting PINK1/Parkin-mediated mitophagy could reduce ROS production and protect against neurons damage (108). Likely, there may be a similar mechanism in cerebral ischemia/reperfusion injury.

#### Mitophagy and Inflammation

Inflammation is an important self-defense mechanism of the body, and mitochondria play a significant role in the occurrence and development of inflammation (109). After being stimulated by factors such as infection, trauma, lipopolysaccharide, high temperature, and hypoxia, the body's innate immune cells activate and trigger an inflammatory response. Under the stimulation of inflammatory factors, ROS and other inflammatory mediators released by neutrophils can induce mitochondrial structural and functional damage (110), including decreased activity of electron transport chain complexes, decreased membrane potential, ATP depletion, and decreased mitochondrial DNA. Mitophagy is of great significance for removing damaged mitochondria and maintaining the function of cellular mitochondrial network. It can remove damaged mitochondria, promote healthy mitochondrial proliferation and other processes, improve mitochondrial homeostasis and function, and exert anti-inflammatory effects (111).

In recent years, a large number of studies have shown that autophagy is inhibited or weakened in inflammatory diseases, and the body is manifested as excessive inflammation or excessive activation of inflammasomes (112–114). And the related mechanisms of mitophagy and inflammation have also been studied in depth (110, 115). In cerebral ischemia/reperfusion injury, the relationship between NLRP3 inflammasome activation and mitophagy has been the most studied. In the rat model of ischemic stroke, studies have found that Parkin-dependent mitophagy could effectively inhibit the activation of NLRP3 inflammasome (80). And in myocardial ischemia/reperfusion injury, activation of PINK1/Parkin-mediated mitophagy could reduce cell apoptosis and inflammatory response (116). At present, the interaction and relationship between the specific mechanisms and pathways of mitophagy and inflammation in ischemic stroke are not fully understood. Uncovering the complex regulatory network mechanism between them can provide a theoretical basis for finding new treatment methods of ischemic stroke.

## Conclusion and Prospects

Obviously, mitophagy plays an important role in ischemic stroke through many regulatory factors and other related cellular processes. Although the role of mitophagy has not been unified yet, most studies have proved that in cerebral ischemia/reperfusion injury, mitophagy as an early defense mechanism could clear damaged mitochondria in time, thereby reducing the further damage to normal mitochondria caused by stimulation. However, when the process of mitophagy is blocked or excessive it will aggravate cerebral ischemia/reperfusion injury. Mitophagy may have different effects on neurons with changes in different pathological stages of ischemia and reperfusion, but the reasons for this change have not been clearly studied. These mechanisms need to be further investigated.

This review summarizes the occurrence and development of mitophagy, the related regulatory factors and signal pathways of mitophagy, and the correlation between mitophagy and other cellular processes after cerebral ischemia/reperfusion, which could help to discover new treatment targets and strategies for ischemic stroke. There are still controversies about the mechanism of mitophagy in cerebral ischemia/reperfusion injury. Exploring mitophagy and its regulation mechanism in cerebral ischemia/reperfusion injury will help to grasp the relationship between mitophagy and cerebral ischemia/reperfusion injury and various diseases, and provide new ideas for clinical treatment. The role of mitophagy in the different stages and cells of cerebral ischemia/reperfusion and the reasons for this change, from beneficial to harmful, need to be further studied.

## Author Contributions

ZS wrote the manuscript and generated the figures. SD helped to design the figures. JZ, HW, and DX proposed suggestions for revisions. CW revised the manuscript. BB and BC designed the content. All authors read and approved the final manuscript.

## Conflict of Interest

The authors declare that the research was conducted in the absence of any commercial or financial relationships that could be construed as a potential conflict of interest.
